# Optimization and Characterization of Interspecific Hybrid Crude Palm Oil Unaué HIE OxG Nanoparticles with Vegetable By-Products as Encapsulants

**DOI:** 10.3390/foods13040523

**Published:** 2024-02-08

**Authors:** Larissa Santos Assunção, Carolina Oliveira de Souza, Fereidoon Shahidi, Tainara Santos Oliveira, Denilson de Jesus Assis, Luis Fernandes Pereira Santos, Itaciara Larroza Nunes, Bruna Aparecida Souza Machado, Camila Duarte Ferreira Ribeiro

**Affiliations:** 1 Graduate Program in Food Science, College of Pharmacy, Federal University of Bahia, Rua Barão de Jeremoabo, 147, Ondina, Salvador 40170-115, Brazil; larissa.sanut@yahoo.com.br (L.S.A.); carolods@ufba.br (C.O.d.S.); tainara@ufba.br (T.S.O.); 2 Department of Biochemistry, Memorial University of Newfoundland, St. John’s, NL A1C 5S7, Canada; fshahidi@mun.ca; 3 Graduate Program in Chemical Engineering (PPEQ), Polytechnic School, Federal University of Bahia, Salvador 40210-630, Brazil; denilson.assis@animaeducacao.com.br; 4 Graduate Program in Food, Nutrition and Health, Federal University of Bahia, Basílio da Gama Street, Rua Basilio da Gama-w/n-Campus Canela, Salvador 40110-907, Brazil; luisfernandes@ufba.br; 5 Graduate Program in Food Science, Department of Food Science and Technology, Federal University of Santa Catarina, Admar Gonzaga Highway, 1346, Itacorubi, Florianópolis 88034-000, Brazil; itaciara.nunes@ufsc.br; 6 Laboratory of Pharmaceutical’s Formulations, SENAI Institute of Innovation (ISI) in Advanced Health Systems (CIMATEC ISI SAS), National Service of Industrial Learning, University Center SENAI CIMATEC, Salvador 41650-010, Brazil; brunam@fieb.org.br

**Keywords:** *Elaeis guineensis*, *Elaeis oleifera*, jackfruit seed, jackfruit central axis, experimental design, nanotechnology

## Abstract

Interspecific hybrid crude palm oil (HCPO) HIE OxG derived from crossbred African oil palm (*Elaeis guineensis*) and American Caiaué (*Elaeis oleifera*) is prominent for its fatty acid and antioxidant compositions (carotenoids, tocopherols, and tocotrienols), lower production cost, and high pest resistance properties compared to crude palm oil. Biodegradable and sustainable encapsulants derived from vegetable byproducts were used to formulate HCPO nanoparticles. Nanoparticles with hybrid crude palm oil and jackfruit seed flour as a wall material (N-JSF) and with hybrid crude palm oil and jackfruit axis flour as a wall material (N-JAF) were optimized using a 2^2^ experimental design. They exhibited nanoscale diameters (<250 nm) and were characterized based on their zeta potential, apparent viscosity, pH, color, and total carotenoid content. The nanoparticles demonstrated a monodisperse distribution, good uniformity, and stability (polydispersity index < 0.25; zeta potentials: N-JSF −19.50 ± 1.47 mV and N-JAF −12.50 ± 0.17 mV), as well as high encapsulation efficiency (%) (N-JSF 86.44 ± 0.01 and N-JAF 90.43 ± 1.34) and an optimal carotenoid retention (>85%). These nanoparticles show potential for use as sustainable and clean-label HCPO alternatives in the food industry.

## 1. Introduction

Crude palm oil (CPO) is extracted from the mesocarp of the fruits of the palm species *Elaeis guineensis* and is a dominant product in oil production globally. CPO production reached 74.7 million tons from the cultivation of 29 million hectares of oil palm in 2020 and increased by 1.9% (18.45 million tons) in 2022 [1]. According to the United States Department of Agriculture (USDA) [2], the leading producers are Indonesia (44.76 million tons), Malaysia (20.14 million tons), and Thailand (2.69 million tons). Brazil ranks seventh among the major oil-producing countries (576.76 tons) [2].

However, owing to the low resistance of CPO to diseases associated with cultivation practices, hybrid crude palm oil (HCPO) started being cultivated. HCPO is obtained from crossbreeding the American and African oil palms (*Elaeis oleifera* and *Elaeis guineensis*, respectively). HCPO contains a higher percentage of unsaturated fatty acids than CPO, and it has a longer shelf life and better pest resistance. The primary producers of this hybrid form are Colombia (accounting for 12% of the total cultivation area for this oil), Ecuador, and Costa Rica [3].

There is an exclusive Brazilian variety of interspecific hybrid (HIE OxG) named Unaué; it is derived from crossbreeding African oil palm (*Elaeis guineensis*) and American Caiaué (*Elaeis oleifera*) and is gaining prominence. HCPO HIE OxG combines the high oil productivity of the oil palm with increased resistance to or tolerance of pests and diseases, particularly the Red Ring disease, a common and highly lethal ailment, in comparison to CPO. Additionally, this variety stands out for producing oil with lower acidity and a milder flavor, and it also has a dwarf stature, leading to lower production costs [4,5]. In the Pará region of Brazil, the cultivated area dedicated to HIE OxG palm oil production is estimated at approximately 11,500 ha, with a potential production value of over 40,000 tons of oil annually [6,7].

HCPO has been explored in the scientific literature for its antioxidant potential, making it a functional oil for disease prevention and health promotion [4,8,9]. Additionally, its prominence is due to its nutritional composition, with high quantities of vitamin E, carotenoids, and unsaturated fatty acids and lower levels of saturated fatty acids [5,10,11]. According to CODEX (2023) [12], this oil contains at least 48% oleic acid and is recognized as “palm oil of higher oleic acid content”. Moreover, this oil has a high carotenoid level, ranging from 500 to 10,000 µg/g, primarily composed of β-carotene (52–60%) and α-carotene (33–36%) [13]. The tocopherol and tocotrienol content of this oil varies from 562 to 1417 µg/g, with γ-tocotrienol being the predominant component (406 to 887 µg/g) [12].

In addition to the nutritional composition of HCPO, it is essential to emphasize that the crude form of this oil is better than its refined form because in the process of refining crude palm oil, notable portions of tocopherols, tocotrienols, and carotenoids may be partially lost, potentially resulting in the formation of carcinogens like 3-monochloropropane-1,2 diol (3-MCPD) and 2-monochloropropane-1,3-diol (2-MCPD) [14]. Moreover, the palm oil sector grew to a market value exceeding USD 50 billion in 2021, with projections indicating a compound annual growth rate (CAGR) of at least 4%. It is anticipated to reach USD 65 billion by 2027 [15].

Despite the potential of this oil as a natural additive owing to its fatty acid and antioxidant (carotenoid, tocopherol, and tocotrienol) composition, the susceptibility of its bioactive compounds to degradation during processing and storage poses a functional loss risk [16]. Nanoencapsulation can increase the stability of bioactive compounds, control their release, and improve them for application in the food industry to, for example, facilitate the incorporation of mainly lipophilic components/nutrients, fortify food, improve oxidative stability, and control microbiological contamination [17]. In this process, small particles of core materials are enclosed within a nanometer-scale wall material (an encapsulant—in this case, one smaller than 1 µm). The selection of the nanoencapsulation method depends on the specific oil being encapsulated and the material used as the encapsulant [18,19]. Additionally, studies have been conducted to develop nano-emulsions containing HCPO for use in packaging, and the physical, thermal, and thermodynamic properties of nano-emulsions have been evaluated [20,21,22,23,24].

Furthermore, for the formation of stable nanoparticles, one must consider the choice of encapsulant, solvent, and emulsifier. In this context, the literature contains studies investigating the development, characterization, and optimization of HCPO nanoparticles as well as the use of different encapsulants [24]. The key independent variables evaluated in the studies include the type and concentration of the encapsulant and the compound to be encapsulated. On the other hand, particle size, polydispersity index, and zeta potential are the main response variables analyzed [23,25].

Additionally, there is an increasing interest in biopolymers and vegetable by-product flours with biodegradable features for encapsulating bioactive compounds and oils [26,27,28]. Non-utilized agriculture by-products, including seed coats, hulls, husks, peels, seeds, and pomace, can precipitate environmental issues. Approximately 40–50% of the gross weight from fruit and vegetable processing is deemed waste. Yet seeds can be used for edible oil extraction, and seed by-products following oil extraction represent roughly 50% of the original seed weight. Despite being rich in vitamins, minerals, nutrients, fibers, and bioactive compounds and potentially suitable for use as functional ingredients and food in the food industry, these by-products are undervalued and typically discarded [29,30,31,32,33,34].

According to the Nanotechnology Products Database [35], there are 423 nanoproducts in 51 different types of food in the food industry, and one food product containing nano-encapsulated oil available on the consumer market was found in the database, namely, a sports nutrition supplement that contains hemp oil, which is promoted for weight loss (ALPHAZS Skinny Tea Tablets). Therefore, nano-encapsulated oils in the food industry represent a crucial area to be explored for the development of new, highly nutritious food products that are innovative, economically viable, and sustainable [17].

The investigation of the nano-encapsulation process of HCPO using vegetable by-products as encapsulants, along with the optimization of formulations and nanoparticle characterization, can facilitate the enhancement and comprehension of fundamental aspects associated with this technology. Therefore, this study aimed to evaluate the most favorable conditions for the nano-encapsulation of HCPO HIE OxG with vegetable by-products as encapsulants. This research could contribute to the development of sustainable and effective strategies for preserving bioactive compounds present in HCPO, with a view to potential applications in the food industry.

## 2. Materials and Methods

### 2.1. Materials

The HIE OxG hybrid fruit was developed by *Empresa Brasileira de Pesquisa Agropecuária* (EMBRAPA) in the Western Amazon (Amazônia, Brazil). The HCPO, known as Unaué, was formulated and provided by the *Comissão Executiva do Plano da Lavoura Cacaueira* (CEPLAC) in the city of Una, Bahia, Brazil. The samples were stored at −20 °C in an amber bottle until the time of analysis. The cowpea shells (*Vigna unguiculata*) used in the preliminary tests were purchased from *Sabor baiano^®^* (Bahia, Brazil). Jackfruit (*Artocarpus heterophyllus*) and yellow passionfruit (*Passiflora edulis* Sims f. *flavicarpa*) were obtained from local markets in Salvador, Bahia, Brazil. Tween 20, Tween 80, and grain alcohol were purchased from Shynth (Diadema, São Paulo, Brazil).

### 2.2. Methods

#### 2.2.1. Preparation of Encapsulants

Passion fruit albedo flour, used in the preliminary tests for nanoparticle formulation, was prepared according to the method reported by Oliveira et al. [36], with modifications. Passion fruits were peeled and washed under running water, and the albedo (mesocarp) was cut into pieces of approximately 1 cm. Subsequently, they were dried in a forced-air oven at 50 °C for 8 h and processed in a knife mill with a 35-mesh sieve. The samples were refrigerated at 4 °C until the time of analysis.

To obtain the cowpea shell flour used in preliminary tests, cowpea shells were thawed and placed in a dehydrator (PE 14 Junior Analogical, Pardal, Petrópolis, Brazil) at 40 °C for a period of 72 h. After drying, the shells were passed through a knife mill (Pulverisette 15, Fritsch, Markt Einersheim, Germany) with a 60-mesh sieve to obtain the flour.

To prepare JSF and JAF, the fruit was used at the green-ripening stage. The seeds and central axes were separated individually from the fruit, followed by blanching (2 min in boiling water at 100 °C and then cooling for 3 min in cold water) and freezing (−10 °C) in a vacuum-sealed package until the day of flour preparation. After thawing under refrigeration (4 °C), the seeds and central axes were dehydrated in a forced-air circulation dehydrator (Q317M-32, Quimis, Diadema, São Paulo, Brazil) at 1.25 m/s^2^ and 50 °C for 36 h. At the end of each drying process, the dehydrated samples were ground in a knife mill (Pulverisette 15, Fritsch, Markt Einersheim, Germany) with a mesh opening of 0.5 mm and vacuum-packed. The samples were refrigerated at 4 °C until the time of analysis.

#### 2.2.2. Preliminary Tests for the Formulation of Hybrid Crude Palm Oil Nanoparticles (N-HCPO)

**Figure 1:**
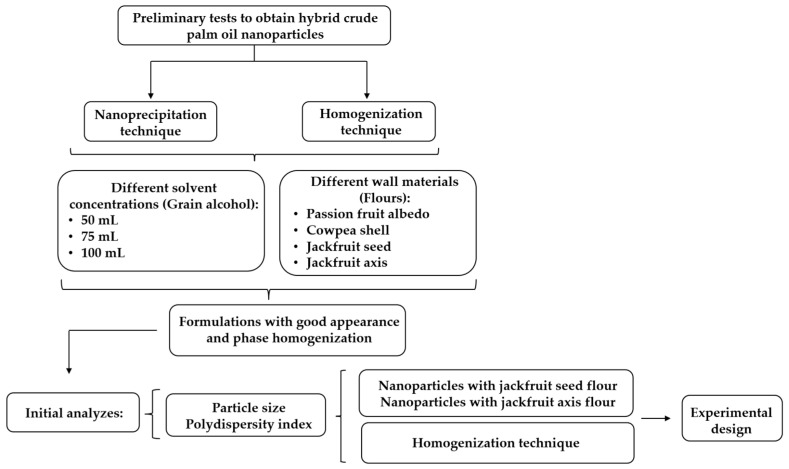
Flowchart of preliminary tests for obtaining N-HCPO. N-HCPO = hybrid crude palm oil nanoparticles; N-JSF = nanoparticles with jackfruit seed flour as the encapsulant; N-JAF = nanoparticles with jackfruit axis flour as the encapsulant.

Preliminary tests were conducted to assess the effects of different techniques, encapsulants, and solvent quantities on N-HCPO production. Various parameters were analyzed, including (a) type of wall material (passion fruit albedo flour, cowpea shell flour, JSF, or JAF), (b) solvent volume (50 mL, 75 mL, and 100 mL), and (c) different techniques (nanoprecipitation and homogenization) (Figure 1).

The homogenization and nanoprecipitation methods used are given below.

The N-HCPO samples were prepared using the homogenization method reported by Ferreira-Ribeiro et al. [14], with some modifications. Tween 20 (250 µL) and HCPO (250 mg) were dissolved in ethyl alcohol (for which different concentrations of the solvent were tested) under agitation (Ika, RH Basic 2, Brazil) for 15 min. This organic phase was then added dropwise to 100 mL of an aqueous solution containing 500 mg of wall material (different flours containing vegetable by-products were tested, which were used individually and not combined) during homogenization using a helical agitator (IKA@, model RW 20 digital, Diagtech, Campinas, São Paulo, Brazil) at a speed of 900 rpm for 30 min. Subsequently, the alcohol was evaporated in a rotary evaporator (35 °C) (Büchi RII, Buchi Brasil Ltda, Valinhos, São Paulo, Brazil) until complete solvent evaporation.

The nanoprecipitation and solvent displacement methods used were adapted from Granata et al. [19]. The organic phase (OP) was prepared by dissolving 310 mg of HCPO in ethyl alcohol, 35 mg of Tween 20, and 90 mg of wall material (different flours containing vegetable byproducts were tested; these wall materials were used individually and not combined), and the resulting mixture was magnetically stirred for 15 min at 1250 rpm (Tecnal TE–085, Piracicaba, São Paulo, Brazil). The OP was then filtered (0.22 μm membrane) and added dropwise to the aqueous phase (AP), containing 50 mL of distilled water and 75 mg of Tween 80, while being kept under magnetic stirring (500 rpm; 10 min) (Tecnal TE–085, Piracicaba, São Paulo, Brazil). Subsequently, the alcohol was evaporated using a rotary evaporator (35 °C) (Büchi RII, Buchi Brasil Ltda, Valinhos, São Paulo, Brazil)

**Figure 2:**
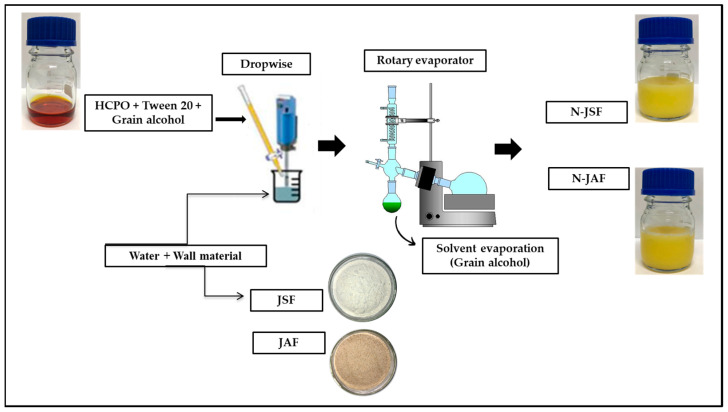
Formulation of N-JSF and N-JAF using the homogenization method. OPBH = hybrid crude palm oil; JSF = jackfruit seed flour; JAF = jackfruit axis flour; N-JSF = nanoparticles with jackfruit seed flour as an encapsulant; N-JAF = nanoparticles with jackfruit axis flour as an encapsulant.

Formulations that exhibited good appearance and phase homogenization were analyzed with respect to particle size and PDI to identify the nanoparticles with the smallest average diameter and monodisperse distribution (Table S1, Supplementary Material). Thus, formulations with jackfruit seed flour (N-JSF) and jackfruit axis flour (N-JAF) as wall materials prepared using the homogenization technique (Figure 1 and Figure 2) were selected and used in the experimental design stage.

#### 2.2.3. Optimization of the Procedure to Obtain N-OPBH Using 2^2^ Factorial Design

Two central composite rotatable designs (CCRD) were used, namely, one with JSF as the wall material and another with JAF, totaling 22 experiments (11 experiments for each design). The experiments were conducted in a randomized manner. Thus, a complete 2^2^ factorial experimental design was developed, with three central points and four axial points at a distance of α = ±1.412. The levels (in coded values) were −1, 0, and +1, where 0 corresponds to the central point.

The design was based on the following independent variables: the amounts of HCPO (200, 250, and 300 mg) and encapsulant (JSF or JAF) (300, 500, and 700 mg). The amounts of solvent (ethyl alcohol), distilled water, and emulsifier (Tween 20) were kept constant during the process. Particle size and PDI were used as experimental responses.

The responses of the variables were analyzed using Statistica Software version 7, and the significance level was set at 5%. The chosen levels were based on preliminary tests.

#### 2.2.4. Particle Size, Polydispersity Index (PDI), and Zeta Potential (ζ)

The particle size, PDI, and zeta potential of N-HCPO were measured using dynamic light scattering and phase analysis light scattering (Zetasizer Nano ZS, Malvern Instruments, Malvern, UK) conducted at 25 °C. Particle size data were reported as the mean diameter and PDI. Zeta potential values were measured based on electrophoretic mobility [37].

#### 2.2.5. Encapsulation Efficiency (EE)

EE was determined using the ultrafiltration/centrifugation technique described by Froiio et al. [38], with modifications. In an Amicon Ultra 0.5/30 kDa filter (Millipore, Carrigtwohill, Ireland), a 500 µL volume of nanoparticles was deposited and then centrifuged at 14,000× *g* for 30 min (Labnet Spectrafuge 24D, Edison, NJ, USA). The sediment obtained after separating the supernatant was diluted in 500 µL of acetone to determine the HCPO content, and this procedure was repeated twice. EE was directly determined using a calibration curve of HCPO in acetone (λ_max_ = 448 nm; Abs = 0.2735 × concentration + 0.014, R^2^ = 0.9908) (Equation (1)):

(1) $$EE=\;\left(\frac{M}{Mo}\right)\times 100$$

where M (mg) is the amount of HCPO loaded into the nanoparticles (determined from the calibration curve), and Mo (mg) is the initial amount of HCPO added to the organic phase for nanoparticle formulation.

#### 2.2.6. Transmission Electron Microscopy (TEM)

The morphology of N-HCPO was determined via Transmission Electron Microscopy (TEM) (TEM; JEOL 1230, Tokyo, Japan). A drop of the nano-emulsion was placed on a grid (Formvar carbon support film, 200 mesh) for 1 min. Subsequently, a drop of a 1% phosphotungstic acid solution was applied for 30 s. The grid was then examined under a transmission electron microscope (TEM; JEOL 1230, Tokyo, Japan) operating at 80 kV with an average magnification of 80,000× and a scale of 100 nm [39].

#### 2.2.7. Apparent Viscosity, pH, Color Parameters, and Total Carotenoids (TC)

The apparent viscosities of free oil and N-HCPO were measured using a concentric cylinder rheometer (Haake Rheotest model 2.1, Medingen, Germany), coupled with a wash bath for temperature control (at 25 °C) and a shear rate of 25–1000 s^−1^. The rheological data were fitted to the Ostwald–de Waele model (Equation (2)) [40].

(2) μ=Kγ(n−1)

Above, µ is the apparent viscosity, K is the consistency index, γ is the shear rate, and n is the flow behavior index. The results are expressed as centipoises (cP).

A commercial pH meter (Sanxin, PHS-3D pH meter, Shanghai, China) was used to determine the nanoparticles’ pH at 25 °C without prior sample dilution and after instrument calibration [14].

The colors of free oil and N-HCPO were determined using a colorimeter CR-400 (Minolta, Osaka, Japan). The data are presented in CIELab Coordinates, which define color in a three-dimensional space with color values (L* (lightness), a* (red/green), and b* (yellow/blue)). The total color difference between the samples was calculated using Equation (3) [25].

∆𝐸 = √ (∆𝐿*)^2^ + (∆𝑎*)^2^ + (∆𝑏*)^2^ (3)

Here, Δ indicates the difference between the parameters among the analyzed samples.

The extraction of oil from the nanoparticles for the determination of total carotenoid content was carried out according to the method reported by Ferreira-Ribeiro et al. [14], with modifications. For this purpose, the N-OPBH was centrifuged with iso-octane and isopropyl alcohol (2:2:1) (SOLAB, SL-706, Piracicaba, Brazil) at 3500 rpm and 25 °C for 10 min a total of 5 times. The obtained supernatants, after consolidation, were filtered through qualitative filter paper 150 mm, added to anhydrous sodium sulfate, and subjected to rotary evaporation of solvents at 35 °C (Büchi RII, New Castle, DE, USA), followed by drying under a stream of nitrogen. The mass of HCPO obtained after extraction was weighed and subsequently diluted in petroleum ether, and the carotenoid content was determined using UV-Vis spectrophotometry, quantifying β-carotene content (λ_max_ = 450 nm; A^1%^_1cm_ = 2592), according to Equation (3) [14]:

(4) $$\mathrm{Total}\;\mathrm{carotenoids}\;\mathrm{content}\;\left(\frac{µg}{g}\right)=A\times \frac{V\left(\mathrm{mL}\right)\times 10^{4}}{A_{1\mathrm{cm}}^{1\%}\times P\left(g\right)}$$

where A is absorbance, V is the total extract volume, P is sample weight, and A^1%^_1cm_ = 2592 (the absorption coefficient of β-carotene in petroleum ether).

#### 2.2.8. Fourier Transform Infrared Spectroscopy (FTIR)

The FTIR spectra of N-JSF, N-JAF, the encapsulants, and HCPO were obtained in absorbance mode using a Perkin Elmer 10 spectrometer operating in the transmission mode region of 4000–400 cm^−1^ and equipped with an attenuated total reflectance (ATR) detector [41]. For the analysis of nanoparticles, the samples were frozen at −80 °C for 24 h and lyophilized (Terroni LS 3000, São Carlos, Brazil) for approximately 72 h to dry them before conducting the analysis.

#### 2.2.9. Statistical Analysis

The analyses were conducted in triplicate (±standard deviation). The means were assessed using analysis of variance (ANOVA) and compared through the Tukey’s test (*p* ≤ 0.05) and Student’s *t*-test (*p* ≤ 0.05) using SAS^®^ OnDemand for Academics software. The results from the experimental design were subjected to statistical tests using Statistica Software (Statsoft, Statistica 7.0, Tulsa, OK, USA).

## 3. Results and Discussion

### 3.1. Optimization of the Procedure for Obtaining N-JSF and N-JAF via 2^2^ Factorial Design

A consensus in the literature regarding the factors that predominantly influence the average diameter of particles and the uniformity of particle size is lacking. Techniques such as nanoprecipitation and homogenization allow the use of different encapsulants, surfactants, and varied concentrations of the organic phase, some of which may interfere with nanoparticles’ characteristics [42,43].

In this context, preliminary tests were conducted to assess the influence of various parameters (different techniques, encapsulants, and solvent volumes) on obtaining hybrid crude palm oil nanoparticles (N-HCPO). Formulations with a good appearance and phase homogenization were analyzed for their particle size and polydispersity index (PDI). According to the results (Table S1, Supplementary Material), N-JSF and N-JAF prepared using the homogenization technique showed smaller particle sizes (*p* < 0.05) than those prepared using the nanoprecipitation technique. According to Fereira-Ribeiro et al. [14], particle size affects the physicochemical properties of a material, compound kinetic release, and biodistribution. Therefore, in future applications, it should be noted that smaller particles tend to better interact with compounds of interest.

According to Ricaurte et al. [21], the smaller the PDI, the lower the tendency for particle aggregation, confirming the quality of the nano-encapsulation method. PDI < 0.25 indicates that the nano-emulsions had a narrow and monodisperse distribution with good uniformity in terms of nanoparticle diameter [14,21]. Only the formulation with passion fruit albedo as a wall material, which was prepared using the homogenization technique, showed a high PDI. The nanoparticles prepared via homogenization with cowpea shell flour, JSF, or JAF showed a PDI ≤ 0.25, with no statistical difference between the samples (*p* > 0.05) (Table S1, Supplementary Material).

With regard to the formulations, when using the nanoprecipitation technique, only nanoparticles with JSF and central axis flour as encapsulants showed a PDI ≤ 0.25. However, these were not used in the experimental design because of their larger particle size compared to those produced using the homogenization technique (*p* < 0.05). Thus, N-JSF and N-JAF, prepared using the homogenization technique, were selected for the experimental design stage considering that, in addition to presenting smaller diameters among all the analyzed formulations, the nano-emulsions had a monodisperse distribution and good uniformity (Table S1, Supplementary Material).

Table 1 shows the particle size and PDI results for N-HCPO with different concentrations of HCPO and wall material evaluated using a 2^2^ factorial design.

**Figure 3:**
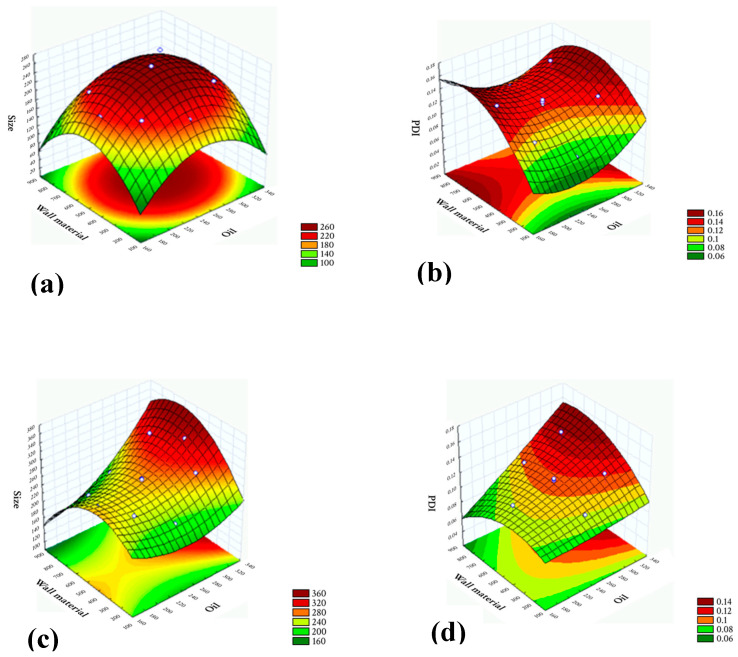
Response surface for the variables amount of oil and wall material with respect to particle size (**a**) and PDI (**b**) for N-JSF as well as with respect to particle size (**c**) and PDI (**d**) for N-JAF.

According to the Pareto diagrams (Figure S1a,b, Supplementary Material) for both experimental designs, the lower the amount of oil and encapsulant added to the formulation, the smaller the particle size. This trend was also evident when analyzing the response surface graphs for the effects of variable amounts of oil and encapsulant on N-JSF (Figure 3a) and N-JAF (Figure 3c) particle sizes. One of the most critical factors affecting the dynamic stability of emulsions is particle size, which influences coalescence. Smaller particles tend to exhibit higher stability, thereby reducing the possibility of coalescence [14].

In addition, the interaction between wall material and oil in the nanoparticle formulation was significant and positive, as indicated by the Pareto plots (Figure S1a,b, Supplementary Material). An increase in the concentration of these variables resulted in larger nanoparticle sizes. Despite this positive interaction, its impact on particle size was smaller than the individual effects of the variables (oil and wall material). According to Table 1, formulation 7, which had the lowest amount of wall material, had the smallest particle size in both experimental designs. This characteristic could be advantageous for the future applicability of nanoparticles, as this formulation has a higher ratio of HCPO:WM (with a mass ratio of 0.8:1; JSF or JAF/HCPO), providing a better potential for greater retention of bioactive compounds.

It is important to note that the amount of oil added to the formulation had a significant and positive effect on particle size (Figure S1a,b, Supplementary Material), similar to wall material. This trend was also observed when analyzing the response surface for the effect of variable amounts of oil and wall material on particle size for N-JSF (Figure 3a) and N-JAF (Figure 3c). As the concentration of oil added to the formulation increased, particle size tended to increase. Formulations 3, 4 (N-JSF and N-JAF), and 6 (N-JAF), which contained higher oil concentrations, also had larger particle diameters (Table 1).

Ricaurte et al. [23] obtained similar results when studying the physical characteristics and thermal and thermodynamic stability of high-oleic palm oil nano-emulsions. The oil concentration was the variable that most affected the response to the analyzed parameters, and higher concentrations of oil led to larger particle sizes. These authors reported that higher oil concentrations resulted in a greater number of dispersed droplets in the aqueous phase, forming macromolecular layers with the encapsulant covered by hydrophilic residues. This process causes the formation of disulfide bonds and thiol/disulphide exchange reactions, leading to the collision of oil droplets and consequently increasing the particle size.

Ricaurte, Perea-Flores, Martinez, and Quintanilla-Carvajal [25] nano-encapsulated high-oleic palm oil (HOPO) by using high-shear homogenization (microfluidization), with whey protein as the wall material and Tween 20 as the emulsifier. In this study, the oil concentration directly influenced particle size, and formulations with higher oil concentrations led to larger particles.

Regarding the PDI, according to the Pareto charts (Figure S2a,b, Supplementary Material), it was noted that the lower the amount of oil and wall material added, the lower the PDI; a similar trend was also observed for particle size. In Figure 3, this trend is also evident when analyzing the response surface for the amount of oil and the encapsulant in the PDI for N-JSF (Figure 3b) and N-JAF (Figure 3d). PDI is a measure that reflects the range of particle size distributions [25]. Values close to 0 indicate that a sample is monodisperse with good uniformity in nanoparticle diameter, whereas values closer to 1 indicate that the sample has a wide range of nanoparticle sizes [44].

According to Hernández-Carrión, Moyano, and Quintanilla-Carvajal [24], the distribution of particles in an emulsion is directly affected by the coalescence and aggregation phenomena of polymers and other constituents present in the wall material, influencing not only the particle size but also the homogeneity with which particles of different sizes are distributed in the aqueous phase. Floury et al. [45] reported that the α and β bonds of secondary structures and the breakage of tertiary and quaternary structures of protein during the nano-encapsulation process can lead to the aggregation of their structures and consequently higher PDI values. The flours used as wall material in this study contained protein and other constituents, such as starch (polysaccharide), in their composition, which may explain the greater tendency for aggregation and, consequently, higher PDI in the formulations with more wall material.

Therefore, considering that a lower PDI value indicates more uniform nanoparticle diameters and a reduced tendency to aggregate, a formulation with lower concentrations of oil and wall material may lead to a reduction in PDI and particle size. In Table 1, formulation 7 (in both experimental designs), with the lowest amount of wall material, yielded the lowest PDI values.

Similar results were reported by Ricaurte, Perea-Flores, Martinez, and Quintanilla-Carvajal [25] and Ricaurte et al. [23]. These authors studied encapsulated HCPO and evaluated the characteristics of nano-emulsions along with the effects of variables through factorial design. The concentrations of wall material and oil were the variables that most affected the PDI, increasing the PDI value as the amounts of these variables increased.

Therefore, considering the best results obtained in the experimental design, formulation 7 was selected and characterized for its zeta potential, encapsulation efficiency, morphology, apparent viscosity, pH, color parameters, and total carotenoid content, as discussed below.

### 3.2. Characterization of N-JSF and N-JAF

#### 3.2.1. Zeta Potential (ζ)

According to Ferreira and Nunes [46], zeta potential indicates the electrical potential of particles and has a significant effect on the stability of a colloidal system. This parameter is strongly influenced by the composition of nanoparticles and the medium in which they are distributed [47]. Zeta potential values above +30 mV and below −30 mV indicate stable suspensions without particle aggregation [48].

N-JSF presented a zeta potential of −19.50 ± 1.47 mV, and N-JAF presented a value equal to −12.50 ± 0.17 mV, indicating good stability of the nano-emulsions and suggesting that repulsive forces were predominant between the droplets in this system [21]. Variations in zeta potential are generally attributed to the chemical characteristics of wall materials [46]. In this study, the negative charges observed may be related to the presence of carboxyl groups in the composition of the flours used as encapsulants [49,50]. Additionally, the emulsifier used in the preparation of the nano-emulsions, Tween 20, generates a negative charge due to the adsorption of hydroxide ions at the oil–water interfaces and the formation of hydrogen bonds between it and the hydroxide ions [51].

Ricaurte et al. [21] reported a zeta potential of −24.8 ± 0.5 mV in nanofibers of HCPO and gelatin. Passion fruit albedo (fruit byproduct) and commercial pectin were used as encapsulants by Bezerra et al. [27] for the production of nanodispersions of carotenoid extract from Spirulina. They reported higher zeta potential values compared to those observed in this study (−41.36 ± 1.43 to −43.64 ± 1.83 mV with passion fruit albedo flour; −24.57 ± 0.66 mV to −27.39 ± 0.86 mV with commercial pectin). The highly negative zeta potential of the nanodispersions with passion fruit albedo flour may be due to the greater number of carboxyl groups that were not replaced by methyl groups, generating more negative charges. Additionally, the encapsulated compound was different from that used in the present study (carotenoid extract).

#### 3.2.2. Encapsulation Efficiency (EE)

The encapsulation efficiency (EE; the amount of oil in nanoparticles) values were 90.43 ± 1.34 and 86.44 ± 0.01% for N-JAF and N-JSF, respectively. EE is related to the stability toward and protection against oxidation [26]. Additionally, EE can vary according to the formulation components, such as oil, emulsifiers, and wall material [26,47]. Values exceeding 80%, as demonstrated in both the studied nanoparticles, revealed that the technology employed for the nano-encapsulation process has a high capacity to protect the oil inside the nanoparticles, suggesting a greater potential for oxidative stability. This result is promising considering the high phytochemical content of HCPO [21].

Sathasivam et al. [41] found an EE of 83–96% in palm oil nanoparticles with carboxymethyl cellulose obtained from sago biomass as an encapsulant. Ferreira-Ribeiro et al. [14] reported EE values of 86.65 ± 1.18–88.13 ± 1.11% in nanoparticles of crude palm oil and its fractions, palm olein, and palm stearin, with casein or gum Arabic used as encapsulants. Ilyasoglu and El (2014) [52] found EE values of 60–80% in fish oil nanoparticles produced through homogenization, and Esfahani et al. [53] reported EE values of 69–98% for different formulations of omega-3 fatty acid nanoparticles with gelatin or gum Arabic using the homogenization technique.

#### 3.2.3. Morphology

**Figure 4:**
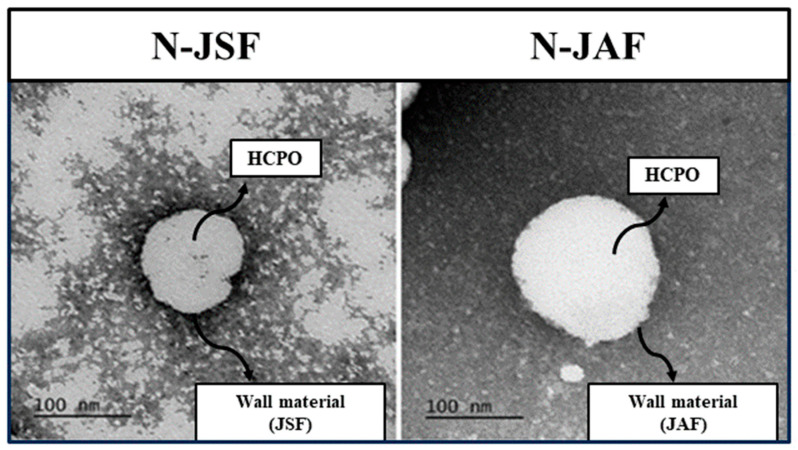
Transmission electron microscopy (TEM) images of a nanoparticle containing hybrid palm oil and jackfruit seed flour as wall material (N-JSF) and a nanoparticle containing hybrid palm oil and jackfruit axis flour as wall material (N-JAF) (scale = 100 nm).

The importance of analyzing the morphology of nanoparticles is related to the comprehensive structural characterization of a material, which allows for the direct observation of dispersed particles. Regardless of the wall material used, the morphologies of N-JSF and N-JAF determined via transmission electron microscopy (TEM) were similar, exhibiting a spherical, regular shape without cracks or aggregates (Figure 4).

Similar results were observed for nanoparticles of chia seed oil [26], crude palm oil and its fractions [14], hybrid palm oil [54], and shrimp oil [55].

#### 3.2.4. Apparent Viscosity, pH, Color Parameters, and Total Carotenoids

The apparent viscosity, pH, color parameters, and total carotenoids (TC) of the nanoparticles are listed in Table 2.

The mean apparent viscosity of the nanoparticles at 25 °C was 21.89 ± 1.20 cP, and no significant difference (*p* > 0.05) was observed among the samples (Table 2). All the analyzed samples exhibited non-Newtonian behavior, indicating that as the shear rate increased, apparent viscosity decreased. Ferreira-Ribeiro et al. [14] prepared nanoparticles of crude palm oil and its fractions, palm olein, and palm stearin using a homogenization technique with casein and gum Arabic serving as encapsulants. They found apparent viscosity values ranging from 14.58 ± 1.22 to 27.10 ± 1.41 cP, similar to the range reported in this study, and the samples also exhibited non-Newtonian behavior.

The rheological characteristics of emulsions can be influenced by the composition and structure of a nano-emulsion (wall material type, concentration, and interaction between the dispersed particles and oil concentration) [56]. Jackfruit seeds have a high content of starch and pectin, and the central axis of the jackfruit contains these constituents [57]. Thus, starch and pectin may have influenced the viscosity of the nano-emulsions. Bezerra et al. [27], using both passion-fruit-albedo-derived pectin and commercial pectin as encapsulants, highlighted that differences in viscosity could be related to various factors, such as particles’ hydrodynamic volume and molar mass and interactions between the wall material, solvent, and encapsulated material. Because of its hydrocolloidal characteristics, pectin tends to contribute to the production of more-viscous emulsions if it contains esterified carbonyl groups.

Ricaurte et al. [23] encapsulated HOPO through microfluidization with the aim of evaluating the physical, thermal, and thermodynamic stabilities of nano-emulsions. One of the parameters assessed was apparent viscosity, which ranged between 1.15 and 80.42 cP. The highest apparent viscosity was obtained for a nano-emulsion with a high whey protein content as an encapsulant, palm oil, and gelatin. The results showed that the concentrations of these constituents significantly affected the viscosity of the emulsion and hence its applicability in the food industry.

Ricaurte, Santagapita, Díaz, and Quintanilla-Carvajal [21] observed the same trend when HOPO was encapsulated using the electrospinning technique. In this study, the diameters and morphologies of the nanoparticles, as well as their physicochemical properties, were investigated. Viscosity values ranged between 64.7 ± 0.1 and 502.1 ± 0.1 cP, and the viscosity increased as the amount of wall material increased. Ricaurte, Perea-Flores, Martinez, and Quintanilla-Carvajal [25] also nano-encapsulated HOPO using high-shear homogenization (microfluidization) using whey protein as a wall material and Tween 20 as an emulsifier; they found apparent viscosity values of 1.9–553.3 cP at 19 °C. Under refrigeration at 4 °C, they reported values of 0.88–112.2 cP over a 4-day storage period. They noted that samples with higher concentrations of whey and oil exhibited higher apparent viscosities.

Viscosity is an important parameter that can influence processing and quality control in the food industry. Depending on the goal of incorporating a nano-emulsion into a food matrix, this parameter can either benefit or detract from the viscosity of the final product. More-viscous food matrices, such as yogurt (viscosity of 35–55 cP), would benefit from the addition of more-viscous nano-emulsions. Conversely, more-fluid matrices such as milk (with a viscosity of approximately 2 cP) would benefit from the addition of less viscous nano-emulsions [14]. As statistical differences between the samples are lacking, it can be suggested that both nanoparticles could be used in the food industry to replace free oil without interfering with the viscosity of the product. For example, yogurt has a viscosity similar to that found in this study (21.89 ± 1.20 cP).

The pH of free oil was 3.52 ± 0.08, indicating acidity, and that of N-JAF was lower (5.49 ± 0.05) than that of N-JSF (5.75 ± 0.05) (*p* < 0.05) (Table 2). However, both formulations had an acidic pH, and nano-encapsulation led to an increase in pH compared to that of the free oil. The difference in pH between free oil and nanoparticles can be explained by the wall materials used. The mean pH of JAF is 5.54 ± 0.29, and it is 5.83 ± 0.06 for JSF [57], constituting values similar to those observed for the nanoparticles.

It is important to note that pH is a crucial indicator of nanodispersion quality and can guide the application of the resultant nanomaterials, especially in food applications. Significantly low pH values indicate strong acidity that can lead to a decrease in the stability of pH-sensitive compounds, such as carotenoids. Additionally, acidity values can also interfere with the taste of a product, and possible changes in pH can indicate the presence of bacteria or chemical reactions, compromising the final quality of a product [39,58]. Thus, owing to their more acidic pH, N-JSF and N-JAF could be used in the preparation of naturally more acidic foods such as yogurt, which usually has a pH of 3.6–4.5, or even in salad dressings, with pH values of 3.2–4.0 [59,60].

According to Campo et al. [26], the pH of a medium influences zeta potential. pH values below 2 tend to favor a slightly positive zeta potential, leading to a reduction in the electrostatic repulsion between particles by reducing groups with similar charges. However, a pH above 2 gradually increases the magnitude of the negative charge, as observed in this study.

As stated in Ferreira-Ribeiro et al.’s study [14], the pH values ranged from 3.82 ± 0.04 to 5.36 ± 0.01 for nanoparticles of crude palm oil and its fractions, palm olein, and palm stearin, similar to those in this study. The more acidic pH reported by the cited authors (3.82 ± 0.04) can be explained by the difference in the wall material used (casein), which has a more amphiphilic characteristic, thereby influencing the reduction in pH.

Considering color parameters, as HCPO is rich in carotenoids, it showed a greater tendency towards red (a* 10.98 ± 0.00) and yellow (b* 11.69 ± 0.01), as confirmed by the color analysis (CIELab) (Table 2). In comparison with African palm oil (*Elaeis guineensis*) analyzed by de Almeida et al. [61], with a value of 20.57 for b*, HCPO showed a less-yellowish color (b* 11.69 ± 0.01). This may be because the hybrid oil had a lower fraction of stearin and a more yellowish palm oil.

The nanoparticles showed a greater tendency towards yellow (higher b* values) than red, and there was no statistical difference between the a* and b* parameters (*p* > 0.05). However, when compared to the free oil, there was a decrease in a* for both nanoparticles (0.64 ± 0.09 N-JSF and 0.55 ± 0.14 N-JAF) and in b* only for N-JSF (8.20 ± 2.53) (*p* < 0.05). Ferreira-Ribeiro et al. [14] encapsulated crude palm oil and its fractions, palm olein, and palm stearin. They reported a* and b* values ranging from 0.52 ± 0.02 to 1.51 ± 0.05 and 5.61 ± 0.07 to 8.36 ± 0.13, respectively, for both fractions analyzed, similar to those in the present study (a* = 0.64 ± 0.09 N-JSF and 0.55 ± 0.14 N-JAF and b* = 8.20 ± 2.53 N-JSF and 10.40 ± 0.39 N-JAF).

The nano-emulsions presented an average L* value of 40.52 ± 2.97, which did not change even with the change in the wall material in the preparation of nanoparticles. This parameter was significantly higher in the nano-emulsions than in the free oil (*p* < 0.05), probably because of the wall materials used (flours present a whitish color), in addition to the water added to the formulation, which can generate a lighter color emulsion.

The total color difference (∆E) between free oil and nanoparticles reflects the distance between two colors, indicating color fading due to its ability to capture all changes in relevant color parameters. This visual threshold is considered significant when the color difference is at least 3 CIELab units, a difference noticeable to a regular observer [62]. Thus, the color change was noticeable in N-JSF (∆E 15.51) and N-JAF (∆E 17.50), attributed to the presence of water in the formulations, resulting in a yellowish color, as indicated in the b* parameter analysis. Furthermore, the free oil exhibited a higher tendency towards red (higher a* values) compared to the nanoparticles (Table 2), which may also explain this color difference.

Color is an important quality attribute in the food industry and serves as the basis for the acceptance of a wide variety of products, positively or negatively influencing the perception of other sensory attributes. Despite the great interest in natural colorants owing to their functionalities, their instability has provoked the industry to invest in synthetic colorants [63]. Therefore, concerning the future applicability of N-JSF and N-JAF in the food industry, these nanoparticles can serve as more stable and promising alternatives for colorants for processed foods due to their protection of the encapsulated pigments, especially carotenoids, which are natural pigments.

The TC content of HCPO before encapsulation was 921.94 ± 28.39 µg/g (Table 2), and this changed to 809.76 ± 41.53 and 799.94 ± 45.60 µg/g for N-JSF and N-JAF, respectively, after encapsulation, constituting a result that is in accordance with the literature findings (500–10,000 µg/g of oil) [13]. Thus, the percentage retention values of carotenoids after encapsulation were 87.83 and 86.77%, respectively, demonstrating the excellent preservation of carotenoids in encapsulated oil compared with that of the free oil. Thus, once encapsulated in nanoparticles, the likelihood of their degradation and consequent loss of functionality is reduced, making them more suitable for application in the food and possibly pharmaceutical industries [64,65]. Therefore, carotenoids and other phytochemicals have been studied for their ability to provide protection against non-communicable chronic diseases (NCDs), wherein oxidative stress is the main contributor [66], and for the development of clean-label food products.

The percentage of carotenoid retention in the nanoparticles with crude palm oil and its fractions, palm olein and palm stearin, was up to 68%, as reported by Ferreira-Ribeiro et al. [14]. This low value may be related to the encapsulation process used (in this case, acetone was used), the extraction method employed, the sensitivity of carotenoids to oxidation, and isomerization during the analysis. However, precautions were taken to achieve their optimal retention in this study.

#### 3.2.5. Fourier Transform Infrared Spectroscopy (FTIR)

**Figure 5:**
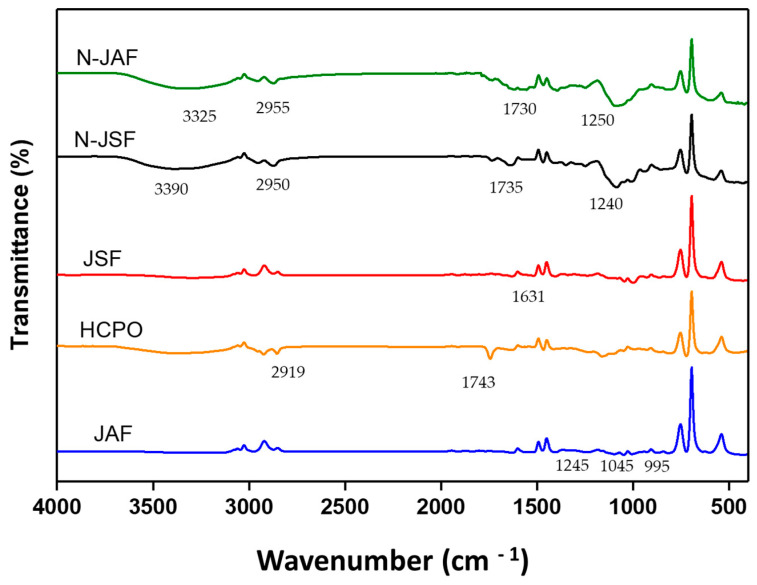
FTIR spectra of nanoparticles containing hybrid palm oil and jackfruit axis flour as wall material (N-JAF); nanoparticles containing hybrid palm oil and jackfruit seed flour as wall material (N-JSF); jackfruit seed flour (JSF); hybrid crude palm oil (HCPO); jackfruit axis flour (JAF).

The FTIR results displaying the characteristic spectra of N-JSF, N-JAF, and the constituents of the nanoparticles (HCPO, JSF, and JAF) are presented in Figure 5.

The spectra were similar among the analyzed samples, and for the nanoparticles (N-JSF and N-JAF), characteristic peaks of the constituents present in the formulations were observed. Thus, the FTIR spectra between the HCPO free oil and the nanoparticles after the oil nano-encapsulation process show no differences. Important functional groups, such as aldehydes (C=O stretching at 1750–1625 cm^−1^, C=O stretching in C-H at 2850–2800 cm^−1^, and C=O stretching in C-H at 2750–2700 cm^−1^), ketones (C=O stretching at 1750–1625 cm^–1^), and carboxylic acid (C=O stretching at 1730–1650 cm^–1^ and O–H stretching bonded by hydrogen at 3400–2400 cm^–1^), were not found in the nanoparticles containing hybrid crude palm oil. This result indicates that no secondary oxidation products were produced during nano-encapsulation. These findings are in line with those reported by Zhang et al. [67] with regard to developing nanoparticles containing palm oil.

The spectra for both the nanoparticles and the free oil revealed that the absorptions in the range of 3000–2800 cm^−1^ were due to C–H stretching vibrations that commonly occur in fats and oils. JSF presented a peak of aromatic C6 ring C=C symmetric stretching at 1631 cm^−1^ [68]. Regarding JAF, the absorption band at 1245 cm^−1^ was attributed to C–O acid stretching. The spectral peaks at 1045 cm^−1^ are due to the presence of the C–O and C–H stretching of sugars such as glucose and sucrose. The spectral peak at 995 cm^−1^ can be attributed to the C–C stretching of sugars such as fructose [69].

## 4. Conclusions

Nanoparticles of HCPO HIE OxG produced via homogenization using a non-toxic solvent (ethyl alcohol) and vegetable by-products as encapsulants demonstrated an appropriate diameter (<250 nm), uniformity (PDI < 0.25), high encapsulation efficiency (>86%), and excellent preservation of carotenoids in the encapsulated oil compared to free oil (>85%). The zeta potential values indicated good stability of the nanoparticles (N-JSF −19.50 ± 1.47 mV, and N-JAF −12.50 ± 0.17 mV), and the morphology demonstrated a spherical and regular aspect for both nanoparticles. The mean apparent viscosity of the nanoparticles was 21.89 ± 1.20 cP, and both formulations presented an acidic pH. Regarding color parameters, there was a higher tendency towards yellow (higher b* value) than red, and the average L* value was 40.52 ± 2.97. These results demonstrate the potential of N-JSF and N-JAF as a clean-label and innovative alternative in oil encapsulation for the preservation of bioactive compounds and application of HCPO. For future perspectives, it is expected that this field can be explored in the development of economically viable, sustainable, and effective edible oil nanoparticles, preserving bioactive compounds, and offering significant nutritional potential for the application of HCPO in the food industry.

## 5. Patents

The patent document “Nanoparticles of Hybrid Crude Palm Oil Unaué HIE OxG (*Elaeis guineensis* × *Elaeis oleifera*) obtained by homogenization method”, stemming from the preliminary tests of this study, has been filed with the National Institute of Industrial Property (INPI) (Process no. BR 10 2022 019533 1) [70].

## Figures and Tables

**Table 1:** 2^2^ factorial design for N-HCPO with jackfruit seed flour or jackfruit axis flour as wall materials, and results obtained from response parameters particle size (nm) and polydispersity index (PDI) responses.

Formulations	Independent Variables		Response Parameters		Response Parameters	
	X1 (HCPO)	X2 (WM)	Size (nm) *	PDI *	Size (nm) **	PDI **
**1**	−1	−1	209.33 ± 2.85	0.10 ± 0.01	250.76 ± 3.27	0.092 ±0.00
**2**	−1	1	204.76 ± 2.98	0.13 ± 0.00	224.80 ± 1.04	0.087 ± 0.00
**3**	1	−1	234.20 ± 2.50	0.13 ± 0.00	283.70 ± 4.92	0.126 ± 0.02
**4**	1	1	241.00 ± 1.49	0.15 ± 0.03	308.20 ± 5.14	0.144 ± 0.01
**5**	−1.41	0	196.90 ± 4.43	0.14 ± 0.01	248.43 ± 1.55	0.108 ± 0.01
**6**	1.41	0	206.50 ± 2.10	0.13 ± 0.02	317.03 ± 2.61	0.128 ± 0.00
**7**	0	−1.41	194.46 ± 0.72	0.07 ± 0.01	212.03 ± 1.25	0.098 ± 0.00
**8**	0	1.41	218.13 ± 2.01	0.12 ± 0.01	230.03 ± 2.37	0.113 ± 0.02
**9 (CP)**	0	0	264.56 ± 1.12	0.12 ± 0.02	266.50 ± 1.25	0.116 ± 0.01
**10 (CP)**	0	0	264.40 ± 3.05	0.12 ± 0.02	266.00 ± 5.57	0.117 ± 0.00
**11 (CP)**	0	0	264.73 ± 4.52	0.12 ± 0.01	265.70 ± 2.33	0.118 ± 0.00

* WM = JSF; ** WM = JAF; CP = central point; HCPO = Hybrid crude palm oil; WM = Wall material; HCPO: −1 = 200 mg; 1 = 300 mg; 0 = 250 mg; −1.41 = 179.28 mg; 1.41 = 320.71. WM: −1 = 300 mg; 1 = 700 mg; 0 = 500 mg; −1.41 = 217.15 mg; 1.41 = 782.84. The results are expressed as the averages of triplicate measurements.

**Table 2:** Apparent viscosity, pH, color parameters, and total carotenoids (TC) of the nanoparticles.

Samples	Apparent Viscosity (cP)	pH	Color Parameters			TC (µg/g)
			L*	a*	b*	
Free oil	23.04 ± 0.18 ^a^	3.52 ± 0.08 ^c^	27.98 ± 0.09 ^b^	10.98 ± 0.00 ^a^	11.69 ± 0.01 ^a^	921.94 ± 28.39 ^a^
N-JSF	22.89 ± 2.38 ^a^	5.75 ± 0.05 ^a^	39.01 ± 3.91 ^a^	0.64 ± 0.09 ^b^	8.20 ± 2.53 ^b^	809.76 ± 41.53 ^b^
N-JAF	20.89 ± 0.82 ^a^	5.49 ± 0.05 ^b^	41.98 ± 0.03 ^a^	0.55 ± 0.14 ^b^	10.40 ± 0.39 ^ab^	799.94 ± 45.60 ^b^

N-JSF = Nanoparticles of HCPO with jackfruit seed flour as an encapsulant. N-JAF = Nanoparticles of HCPO with jackfruit axis flour as an encapsulant. The data are expressed as means ± standard deviation (n = 3). Different letters in the same column indicate significant differences (*p* < 0.05).

## Data Availability

The original contributions presented in the study are included in the article/supplementary material, further inquiries can be directed to the corresponding author.

## Supplementary Materials

The following Supporting Information can be downloaded from https://www.mdpi.com/article/10.3390/foods13040523/s1. Table S1: Results of preliminary tests for the development of N-HCPO with different wall materials; Figure S1: Pareto diagram concerning the effect of the amount of oil and wall material on particle size for N-JSF (a) and N-JAF (b); Figure S2: Pareto diagram concerning the effect of the amount of oil and wall material on PDI for N-JSF (a) and N-JAF (b).
