# Supplementary material for: Optimization and Characterization of Interspecific Hybrid Crude Palm Oil Unaué HIE OxG Nanoparticles with Vegetable By-Products as Encapsulants
Source: Foods. 2024 Feb 8;13(4):523. doi: 10.3390/foods13040523 (PMC10887919; doi:10.3390/foods13040523)
Supplement: Supplementary file 1 [file foods-13-00523-s001.zip › foods-2802409-supplementary.pdf]

Supplementary material

Article

Optimization and characterization of hybrid crude palm oil Unaué HIE OxG nanoparticles with vegetable by-products as encapsulants

Larissa Santos Assunção [1], Carolina Oliveira de Souza [1], Fereidoon Shahidi [2], Tainara Santos Oliveira [1], Denilson de Jesus Assis [3], Luis Fernandes Pereira Santos [4], Itaciara Larroza Nunes [5], Bruna Aparecida Souza Machado [6] and Camila Duarte Ferreira Ribeiro [1,4,*]

[1] Graduate Program in Food Science, College of Pharmacy, Federal University of Bahia, Rua Barão de
Jeremoabo, 147, Ondina, Salvador 40170-115, Brazil; larissa.sanut@yahoo.com.br (L.S.A.); carolods@ufba.br (C.O.d.S.); tainara@ufba.br (T.S.O.)

[2] Department of Biochemistry, Memorial University of Newfoundland, St. John's, NL A1C 5S7, Canada;
fshahidi@mun.ca

[3] Graduate Program in Chemical Engineering (PPEQ), Polytechnic School, Federal University of Bahia,
Salvador 40210-630, Brazil; denilson.assis@animaeducacao.com.br

[4] Graduate Program in Food, Nutrition and Health, Federal University of Bahia, Basílio da Gama Street,
Rua Basilio da Gama-w/n-Campus Canela, Salvador 40110-907, Brazil;
luisfernandes@ufba.br

[5] Graduate Program in Food Science, Department of Food Science and Technology, Federal University of Santa Catarina, Admar Gonzaga Highway, 1346, Itacorubi, Florianópolis 88034-000, Brazil;
itaciara.nunes@ufsc.br

[6] Laboratory of Pharmaceutical's Formulations, SENAI Institute of Innovation (ISI) in Advanced Health
Systems (CIMATEC ISI SAS), National Service of Industrial Learning, University Center SENAI CIMATEC, Salvador 41650-010, Brazil; brunam@fieb.org.br

* Correspondence: camiladuartef@ufba.br; Tel./Fax: +55-71-99132-0655

Table S1. Results of preliminary tests for the development of N-HCPO with different wall materials.

Technique	Wall material (mg)	HCPO (mg)	Ratio (WM: HCPO)	Size	PDI
**Homogenization (Ferreira-Ribeiro et al. 2022)**	Passio fruit albedo (500)	250	2:1	466.00 ± 12.00^{aC}	0.34 ± 0.03^{aB}
	Cowpea shell (500)	250	2:1	292.56 ± 3.43^{bCD}	0.10 ± 0.01^{bC}
	**Jackfruit seed (500) ***	**250**	**2:1**	**247.63 ± 4.13^{cD}**	**0.09 ± 0.02^{bC}**
	**Jackfruit axis (500) ***	**250**	**2:1**	**224.20 ± 1.60^{dD}**	**0.13 ± 0.01^{bC}**
**Nanoprecipitation (Granata et al. 2018)**	Passio fruit albedo (90)	310	0,3:1	1068.18 ± 233.98^{aA}	0.73 ± 0.18^{aA}
	Cowpea shell (90)	310	0,3:1	699.73 ± 27.15^{bB}	0.40 ± 0.06^{bB}
	Jackfruit seed (90)	310	0,3:1	364.20 ± 9.00^{cC}	0.11 ± 0.03^{cC}
	Jackfruit axis (90)	310	0,3:1	324.45 ± 5.43^{cC}	0.06 ± 0.03^{cC}

*Best results.

The data are expressed as mean ± standard deviation (n = 3).
Mean values with different lower-case letters indicate significant differences ($p < 0.05$) within the same column (same size or PDI) for the same technique.
Mean values with different capital letters indicate significant differences ($p < 0.05$) between homogenization and nanoprecipitation techniques.

(a) **(b)**

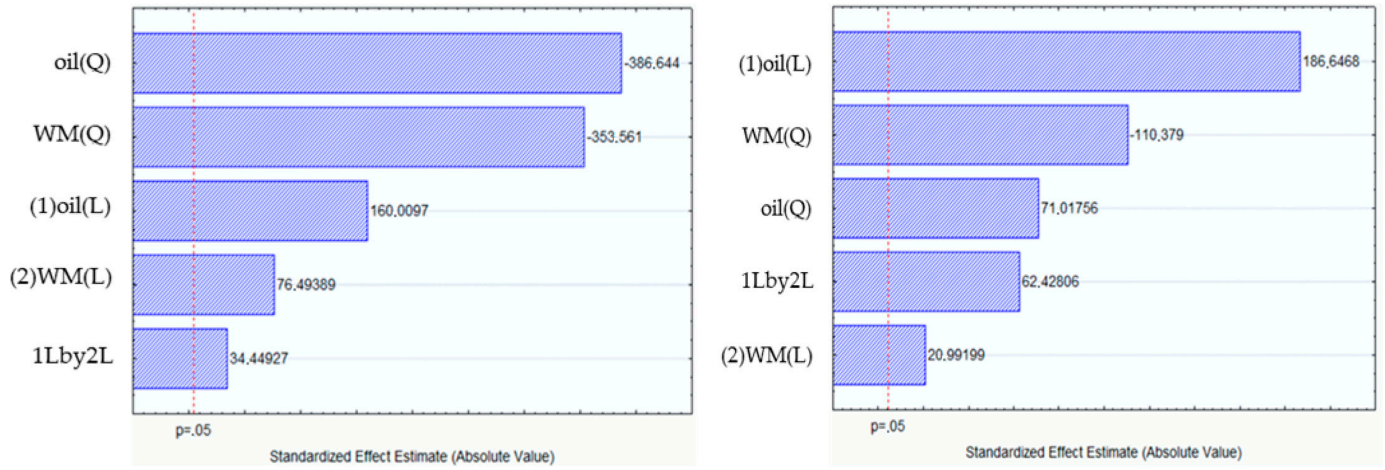

Figure S1. Pareto diagram for the variables amount of oil and wall material on particle size for N-JSF (a) and N-JAF (b).

(a) **(b)**

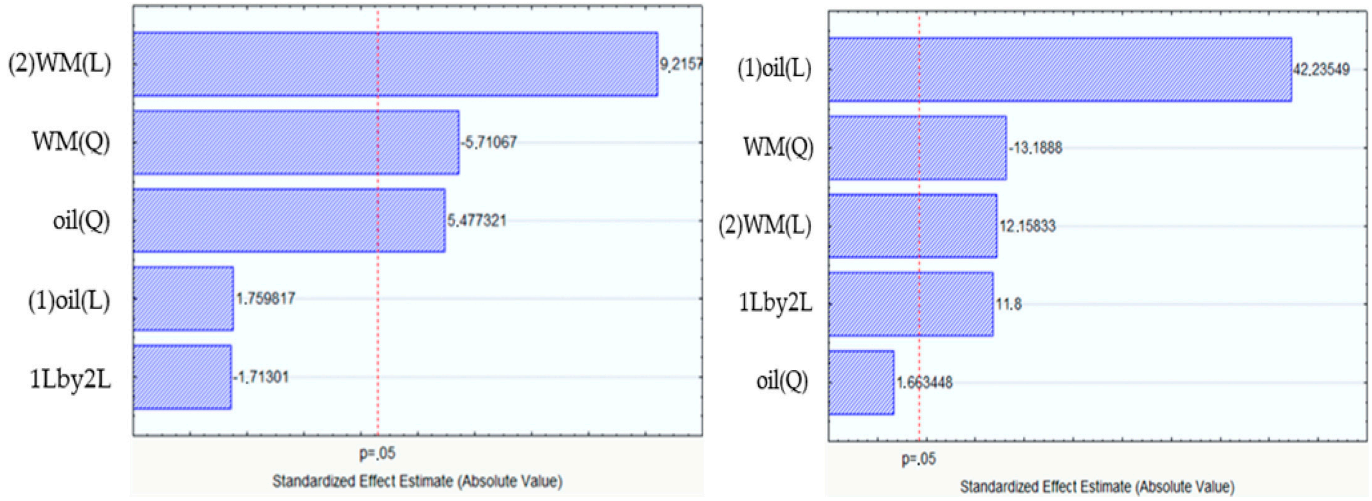

Figure S2. Pareto diagram for the variables amount of oil and wall material on PDI for N-JSF (a) and N-JAF (b).
